# Pacemaker Malfunction in a Patient With Congestive Heart Failure and Hypertension

**DOI:** 10.7759/cureus.34574

**Published:** 2023-02-02

**Authors:** Abdullah Saeed, Abdullah AlShafea, Foton A., Abdulrahman Bin Saeed

**Affiliations:** 1 Action Research, Ministry of Health, Abha, SAU; 2 Resarch Unit, Ministry of Health, Abha, SAU; 3 Public Health, King Khalid University, Khamis Mushait, SAU; 4 Public Health, King Abdulaziz University, Khamis Mushait, SAU

**Keywords:** chatgpt, hypertension, malfunction, pacemaker complication, heart failure, pacemaker

## Abstract

A pacemaker is a medical device commonly used to regulate a patient’s heartbeat in cases where the heart’s natural electrical impulses may be erratic or compromised. Failure of a pacemaker, or pacemaker malfunction, can be life-threatening and requires immediate action to prevent serious complications. This case report describes a 75-year-old male patient with a history of ventricular tachycardia, congestive heart failure, hypertension, and smoking who was admitted to the hospital with symptoms of palpitations, dizziness, lightheadedness, and decreased level of alertness. The patient had a single-chamber pacemaker implanted two years prior to the current admission. Upon physical examination, the patient’s pacemaker had failed, and he was diagnosed with pacemaker failure. Differential diagnoses were ranked from most likely to least likely, based on the patient’s history and physical examination, and included pacemaker failure, arrhythmia, myocardial infarction, and pulmonary embolism. Treatment included the replacement of the pacemaker, and the patient was discharged in stable condition.

## Introduction

A pacemaker is a small device that is implanted under the skin of the chest to help regulate heartbeat [1]. Pacemaker malfunction refers to any deviation from the normal function of the device that can lead to significant cardiac problems. This can include battery failure, lead displacement or fracture, or software issues [2]. Pacemaker malfunction can result in symptoms such as dizziness, fainting, and chest pain [3]. In severe cases, it can lead to cardiac arrest [4]. Another study found that the risk of pacemaker malfunction is higher in patients who have multiple leads or those who have had previous surgeries to replace or repair their pacemakers [5]. In case of pacemaker malfunction, it is important to seek medical attention immediately, as it can be life-threatening [6]. Treatment options may include pacemaker replacement or repair, or the use of an external pacemaker until the implantable device can be fixed [7].

ChatGPT is an artificial intelligence tool that was used to generate the text that was used as a helpful aid in conducting this case report (Appendices).

## Case presentation

This case study shows how a pacemaker failure was detected and managed. The patient is a 75-year-old male who has had ventricular tachycardia, congestive heart failure, and hypertension in the past. He had a smoking habit growing up. The patient had also had a single-chamber pacemaker implanted two years prior to the current admission.

Clinical manifestations

**Figure 1:**
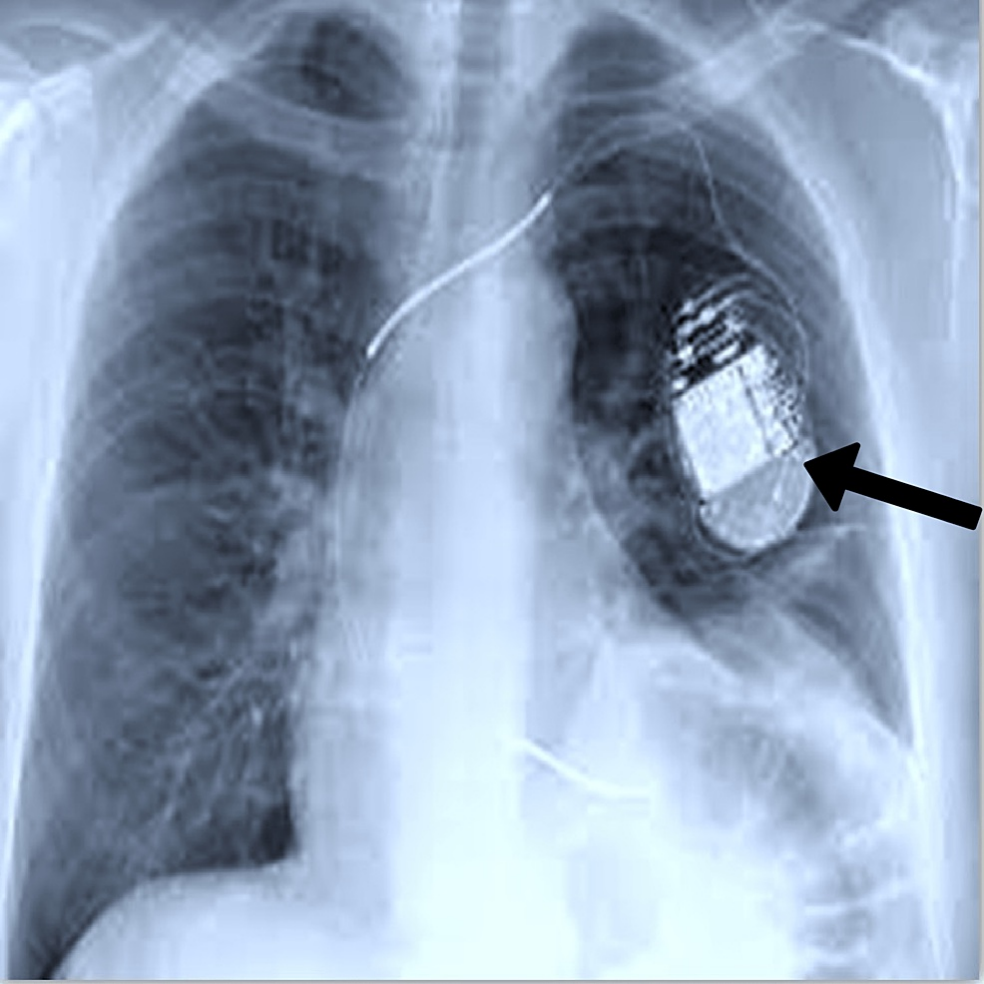
X-ray showing the pacemaker in position (arrow)

The patient was admitted to the hospital with palpitations, dizziness, lightheadedness, and a loss of alertness. His pulse was irregular during the physical examination. The electrocardiogram (ECG) showed a sinus rhythm with intermittent capture failure, indicating a pacemaker malfunction. To control and stabilize the arrhythmia, the patient was started on an intravenous (IV) infusion of amiodarone. The pacemaker settings were adjusted, and an echocardiogram revealed normal systolic function and a normal left heart ejection fraction of the ventricle. The patient was sent home with instructions to contact his cardiologist. The pacemaker is shown in position in Figure 1.

**Figure 2:**
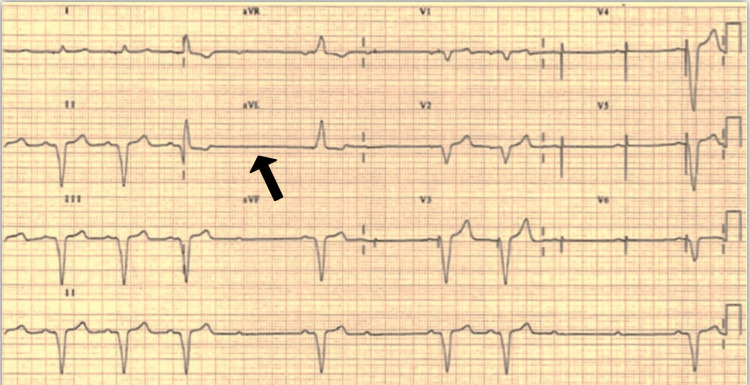
Pacing failure: ECG showing intermittent failure to capture (arrow)

Figure 2 shows pacing failure. The ECG demonstrates a ventricular-paced rhythm with intermittent capture failure. Following each P wave, atrial sensing appears to be intact ventricular pacing spikes, most easily seen in V3-6 (tiny pacing spikes are also visible in I, aVR, and V1).

## Discussion

Intermittent failure to capture is a condition that occurs when the myocardium does not respond to the electrical stimuli from the pacemaker or implantable cardioverter-defibrillator (ICD); this condition is referred to as “loss of capture,” also known as “non-capture.” It can result from various conditions, such as a low battery, interference from external electromagnetic sources, or mechanical issues in the pacemaker. The symptom of an intermittent failure to capture is typically a feeling of dizziness or fatigue. It is important to note that a pacemaker is an electrical device that regulates the heart’s rhythm, and the sources of intermittent failures can vary.

One of the most common causes of intermittent failure to capture is device failure. It is possible that the electricity stored in the capacitors needs to be properly recharged during a battery change or when a device is upgraded. Another possibility is that the battery connection is loose and not providing the necessary power for proper functioning. Environmental factors or electromagnetic interference can also be sources of intermittent failure. For example, radio frequency identification (RFID) or magnetic resonance imaging (MRI) scanners are known to interfere with pacemakers. Additionally, it is possible that some external devices, such as a defibrillator or a device that transmits electrical signals, can interfere with the pacemaker [8]. Finally, the patient’s body can also be a source of intermittent failure. This can happen if the patient has a diseased or impaired heart, in which case insufficient voltage may be available for the pacemaker to provide pulses effectively. Other issues, such as a decrease in the patient’s blood pressure, may also lead to intermittent failure to capture. Intermittent failure to capture is an important and potentially serious condition. Identifying the source of the failure to establish the right treatment approach is essential. If the cause is environmental, proper avoidance of disruptive sources should be taken.

Appropriate medication and lifestyle changes should be implemented if it is related to the patient’s health. Regardless of the root cause, it is crucial to visit a medical professional to ensure that the pacemaker is functioning correctly [9]. A pacing spike can be seen on the electrocardiogram or rhythm strip, but there is no subsequent P or QRS complex that illustrates this situation, where the atrial pacing stimuli do not capture the atrial tissue, and there is no subsequent atrial depolarization with P waves. Without a proper capture of the chamber being paced, there may be a sign of pacing on the near- or far-field electrocardiogram during a device interrogation. The timing of the implant strongly correlates with particular causes (especially immediately post-implantation), and there are numerous causes for the loss of capture [10].

## Conclusions

In this case report, the patient’s pacemaker malfunction was successfully managed with IV amiodarone to help reduce the rate of the ventricular arrhythmias and adjustment of the pacemaker settings to restore a normal rhythm. Through tracking of electrical signals and measurements, pacemaker function was indicated as proper. It is important to recognize the signs and symptoms of pacemaker malfunction and take immediate action to prevent serious complications.

## Figures and Tables

**Figure 3:**
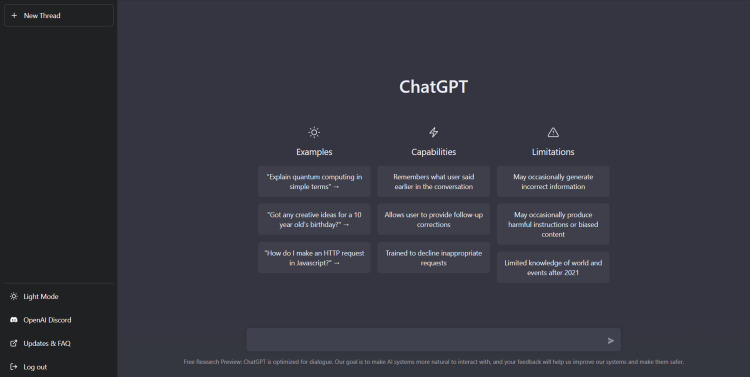
ChatGPT

## Appendices

ChatGPT, an artificial intelligence tool, was used to generate the text in this case report (Figure 3).
